# Evaluation of the efficacy of peripheral nerve block alone in episodic and chronic migraine patients

**DOI:** 10.1055/s-0043-1771494

**Published:** 2023-08-03

**Authors:** Gokhan Evcili, Ahmet Yabalak

**Affiliations:** 1 University of Health Sciences, Derince Training and Research Hospital, Department of Neurology, Kocaeli, Turkey.; 2 Duzce University, Faculty of Medicine, Department of Neurology, Duzce, Turkey.

**Keywords:** Migraine Disorders, Nerve Block, Headache, Transtornos de Enxaqueca, Bloqueio Nervoso, Cefaleia

## Abstract

**Background** Peripheral nerve block (PNB) is usually performed in patients with migraine who are resistant to treatment with medications.

**Objective** To compare the efficacy of PNB alone and PNB combined with prophylactic medications in migraine patients.

**Method** The data on migraine patients who underwent PNB in our clinic between November 2019 and January 2022 were retrospectively reviewed. Blocks of the greater occipital nerve (GON), lesser occipital nerve (LON) and supraorbital nerve (SON) were performed upon admission and in the second week.

**Results** The study included 116 patients. While 21 out of 39 episodic migraine (EM) patients continued to use prophylactic medications, 18 were followed up with PNB alone. While 49 out of 77 chronic migraine (CM) patients continued to use prophylactic medications, 28 were followed up with PNB alone. Comparison of the admission and second-month data of the patients who only underwent PNB and those who continued the drug treatment together with PNB in both the EM and the CM group showed that the number of days with pain, number of analgesics taken and scores on the Visual Analog Scale (VAS) and the Migraine Disability Assessment (MIDAS) were significantly reduced in both groups ( *p* < 0.01). Comparison of the second-month data of the patients followed up with PNB alone and those followed up with PNB together with prophylactic medications showed that there was no significant difference between the EM and CM patients ( *p* > 0.05).

**Conclusion** Bilateral GON, LON and SON block with lidocaine injection seems to be an effective treatment on its own, without the need for prophylactic medications, in both EM and CM patients during a two-month follow-up.

## Resumo

**Antecedentes** O bloqueio de nervos periféricos (BNP) geralmente é realizado em pacientes com migrânea resistentes ao tratamento medicamentoso.

**Objetivo** Comparar a eficácia do BNP isolado e do BNP combinado com medicamentos profiláticos em pacientes com enxaqueca.

**Método** Os dados de pacientes com enxaqueca submetidos a BNP em nossa clínica entre novembro de 2019 e janeiro de 2022 foram revisados retrospectivamente. Bloqueios do nervo occipital maior (NOM), nervo occipital menor (NOM) e nervo supraorbital (NSO) foram realizados na admissão e na segunda semana.

**Resultados** O estudo incluiu 116 pacientes. Enquanto 21 dos 39 pacientes com enxaqueca episódica (EE) continuaram a usar medicamentos profiláticos, 18 foram acompanhados apenas com BNP. Enquanto 49 dos 77 pacientes com enxaqueca crônica (EC) continuaram a usar medicamentos profiláticos, 28 foram acompanhados apenas com BNP. A comparação dos dados de admissão e do segundo mês dos pacientes que fizeram apenas BNP e daqueles que continuaram o tratamento medicamentoso junto com BNP, tanto no grupo EE quanto no grupo EC, mostrou que o número de dias com dor, o número de analgésicos tomados e os escores da Escala Visual Analógica (EVA) e da Avaliação da Incapacidade da Enxaqueca (AIE) foram significativamente reduzidos em ambos os grupos (p < 0,01). A comparação dos dados do segundo mês dos pacientes acompanhados apenas com BNP e aqueles acompanhados com BNP juntamente com medicações profiláticas mostrou que não houve diferença significativa entre os pacientes EE e EC (p > 0,05).

**Conclusão** O bloqueio bilateral de NOM, NOM e NSO com injeção de lidocaína parece ser um tratamento eficaz por si só, sem a necessidade de medicamentos profiláticos, tanto em pacientes EE quanto com EC durante um seguimento de dois meses.

## INTRODUCTION

Migraine is a well-defined primary headache syndrome with a prevalence of 17.6% in women and 5.7% in men. 1 2

This disease, which is quite common in society, results in disability for the patients and costs for society through drug use and loss of workforce. Although there are many medical treatment options for reducing the frequency of attacks in migraine patients, treatment compliance is very low due to the need for long-term drug use and due to intolerable side-effect profiles. It has been observed 3 that 28.3% of episodic migraine (EM) patients and 44.8% of chronic migraine (CM) patients use regular medical treatments. Apart from medical treatments, injections of botulinum toxin have also been used for treating migraine. However, the need for multiple injections in one session, the need for repetitive injections and the high costs limit their use. 4

Peripheral nerve block (PNB) has long been used for treating migraine. Although variations in the nerves blocked, frequency of use of PNB and anesthetic agents applied have been reported, this procedure has been found to be effective for treating migraine in many studies. 5 6

While most studies have been conducted on CM patients, the number of studies on the efficacy of PNB in EM patients is more limited. 4 6 7 8 Almost all of the latter studies included patients who did not respond to drug treatment, and the patients who underwent PNB continued to use prophylactic medications. 4 5 6 Only one study 9 compared patients who underwent greater occipital nerve (GON) block with and without drug treatment, and this found that there was no difference in efficacy of treatment between the two groups. In the literature, while there are studies in which GON block alone was performed, there are others in which it was performed together with supraorbital nerve (SON) block, which has also been found to be effective. 5 6 8 10 11 In addition, while the effects of PNB on the frequency and severity of pain have been evaluated in studies, the number of studies evaluating the effect on the migraine disability score is limited. 4 5 6

We also perform PNB on migraine patients in our clinic, and in our previously published study, 12 in line with data in the literature, we found that it was an effective treatment option. In our clinic, we have sometimes stopped use of prophylactic medications before PNB in some patients. However, we have only found one study in the literature on the efficacy of PNB for migraine prophylaxis without drug treatment: Inan et al. 9 observed that there was no difference in terms of efficacy between GON block alone and in combination with the drug treatment. However, those authors 9 did not report how many patients had EM and how many had CM. In the present study, we aimed to compare and evaluate the effectiveness of PNB alone and in combination with prophylactic medications in both EM and CM patients.

## METHODS

In the present retrospective study, after obtaining approval from the Ethics Committee of the Derince Training and Research Hospital (2022-41, May 26, 2022), we reviewed the files of patients diagnosed with EM and CM in accordance with the International Classification of Headache Disorders, third edition 2 (ICHD-3), who underwent PNB in our clinic between November 2019 and January 2022. Patients older than 18 years of age, diagnosed with migraine for at least 1 year, using the same prophylactic treatment for at least 3 months and having a score on the Visual Analog Scale (VAS) > 5, were included in the study. Patients who did not come for their second injection, those who did not come to the follow-ups and those who were addicted to alcohol and other substances were excluded. We evaluated the data of 116 patients who met these criteria, among whom 46 were treated with PNB after discontinuing their prophylactic medication treatment and 70 underwent PNB in combination with their prophylactic treatment. The prophylactic treatments were discontinued at least two weeks before the injection in patients followed up with PNB alone.

### Procedure

The patients underwent bilateral GON, lesser occipital nerve (LON) and SON blocks upon admission and in the second week. First, the area to be injected was wiped with an antiseptic solution. Then, 1.5 mL of 2% lidocaine was injected 2 cm laterally and 2 cm inferiorly to the occipital protuberance for GON block. The LON block was performed by injecting 1.5 mL of 2% lidocaine at the 2/3 lateral point on a line between the occipital protuberance and the mastoid; and the SON block was performed by injecting 1 mL of 2% lidocaine just above the supraorbital notch. A 27-G needle was used in all injections. The patients were monitored for 30 minutes after the procedure to watch for early side effects. Two weeks later, the same protocol was repeated. The VAS scores, number of days in pain, number of analgesics taken in the previous month and Migraine Disability Assessment (MIDAS) scores at baseline and at 2 months were noted. 13 Dexketoprofen and frovatriptan were prescribed to patients as rescue therapy during migraine attacks.

The primary outcome was determined in terms of the decrease in the severity of pain and the number of days in pain, and the secondary outcome consisted of the decrease in the MIDAS scores.

### Statistical analysis

All data were analyzed using the Statistical Package for the Social Sciences (IBM SPSS Statistics for Windows, IBM Corp., Armonk, NY, United States) software, version 21.0. The data were expressed as numbers, means, standard deviations and medians. For normally-distributed data, an independent-sample *t* test was used to evaluate independent groups and paired-sample *t* tests were used to evaluate repeated measurements. For data that did not show normal distribution, the Mann-Whitney U test was used to evaluate independent groups and the Wilcoxon test was used to evaluate repeated measurements. Values of *p* < 0.05 were considered statistically significant.

## RESULTS

A total of 116 patients were included in the study. Out of the 39 EM patients, 21 underwent PNB while continuing to take medications (EM M + PNB group) and 18 underwent PNB alone (EM PNB group) after their prophylactic medication treatment had been discontinued. Out of the 77 CM patients, 49 composed the medication plus PNB (CM M + PNB) group and 28 formed the group with PNB alone (CM PNB).

Evaluation of the data on all the migraine patients regarding the number of days in pain and the number of analgesics taken in the previous month showed that the VAS and MIDAS scores of both the PNB and the M + PNB group were significantly lower than the values found upon admission ( *p* < 0.01). Comparison of the second month data of the two groups did not show any further significant difference ( *p* > 0.05) ( Table 1 ).

**Table 1:** Comparison of the demographics and treatment efficacy data of the migraine patients included in the study

	Peripheral nerve block group (n = 46)	Drug treatment + peripheral nerve block group (n = 70)	*p* -value
**Age (years)**	41.24 ± 11.91	39.26 ± 9.73	0.32*
**Disease duration (years)**	12.8 ± 8.76	13.0 ± 6.85	0.89*
**Sex: Male/Female**	7/39	10/60	0.89 ^&^
**VAS score upon admission**	7.91 ± 0.93	8.09 ± 0.69	0.25*
**VAS score after 2 months**	4.72 ± 2.69	5.37 ± 2.66	0.19*
***p*** **-value**	0.00 ^#^	0.00 ^#^	
**MIDAS score upon admission**	37.02 ± 13.24	39.79 ± 12.54	0.25*
**MIDAS score after 2 months**	12.41 ± 10.91	11.9 ± 11.43	0.81*
***p*** **-value**	0.00 ^#^	0.00 ^#^	
**Number of analgesics taken upon admission**	17.67 ± 11.87	17.91 ± 9.5	0.9*
**Number of analgesics taken after 2 months**	4.13 ± 5.84	6.34 ± 8.36	0.12*
***p*** **-value**	0.00 ^#^	0.00 ^#^	
**Number of days with pain upon admission**	18.59 ± 9.2	19.13 ± 8.25	0.74*
**Number of days in pain after 2 months**	7.41 ± 8.62	7.47 ± 9.02	0.97*
***p*** **-value**	0.00 ^#^	0.00 ^#^	

Abbreviations: MIDAS, Migraine Disability Assessment; VAS, Visual Analog Scale.

Notes: ^*^ independent-sample *t* test; ^#^ paired-sample *t* test; ^&^ chi-square test.

Comparison of all the migraine patients in terms of disease duration, patient age, VAS and MIDAS scores upon admission, number of days in pain and number of analgesics taken in the previous month showed that disease duration was significantly longer in the EM M + PNB group than in the EM PNB group ( *p* = 0.02). There was no significant difference between the groups in terms of other demographic or baseline clinical data.

Evaluation of the EM and CM patients separately showed that the VAS scores in the second month, number of analgesics taken in the previous month, MIDAS scores and number of days in pain were significantly lower in both groups, regardless of the treatment ( *p* < 0.01). Comparison of the data pertaining to the second month showed that there was no significant difference between the EM and CM patients, regardless of the treatment ( *p* > 0.05) ( Table 2 ).

**Table 2:** Evaluation of the demographics and treatment efficacy data among episodic and chronic migraine patients

	EM M + PNB group (n = 21)	EM PNB group (n = 18)	*p* -value	CM M + PNB group (n = 49)	CM PNB group (n = 28)	*p* -value
**Age (years)**	38.43 ± 7.62	37.22 ± 10.46	0.56*	39.61 ± 10.56	43.82 ± 12.24	0.11 ^$^
**Disease duration (years)**	14.29 ± 6.56	9.33 ± 7.52	0.02*	12.45 ± 6.96	15.04 ± 8.9	0.16 ^$^
**Sex: Male/Female**	6/15	3/15	0.37 ^£^	4/45	4/24	0.39 ^£^
**VAS score upon admission**	8.05 ± 0.66	7.56 ± 0.98	0.07 ^#^	8.1 ± 0.71	8.14 ± 0.84	0.82 ^$^
**VAS after 2 months**	5.19 ± 3.10	4.83 ± 2.81	0.58 ^#^	5.45 ± 2.48	4.64 ± 2.57	0.18 ^$^
***p*** -value	0.001*	0.001*		0.00 ^&^	0.00 ^&^	
**MIDAS score upon admission**	29.10 ± 5.90	27.61 ± 5.46	0.49 ^#^	44.37 ± 11.84	43.07 ± 13.28	0.66 ^$^
**MIDAS after 2 months**	8.43 ± 6.48	7.72 ± 4.75	0.9 ^#^	13.39 ± 12.76	15.43 ± 12.66	0.50 ^$^
***p*** **-value**	0.00*	0.00*		0.00 ^&^	0.00 ^&^	
**Number of analgesics taken upon admission**	9.48 ± 5.65	9.11 ± 4.25	1 ^#^	21.53 ± 8.49	23.18 ± 11.97	0.48 ^$^
**Number of analgesics taken after 2 months**	3.67 ± 4.93	3.39 ± 3.44	0.79 ^#^	7.49 ± 9.26	4.61 ± 6.98	0.15 ^$^
***p*** **-value**	0.003*	0.001*		0.00 ^&^	0.00 ^&^	
**Number of days in pain upon admission**	10.1 ± 2.21	9.22 ± 2.01	0.16 ^#^	23.0 ± 6.69	24.61 ± 6.52	0.31 ^$^
**Number of days in pain after 2 months**	4.71 ± 5.01	3.94 ± 3.45	0.98 ^#^	8.65 ± 10.09	9.64 ± 10.15	0.68 ^$^
***p*** **-value**	0.002*	0.001*		0.00 ^&^	0.00 ^&^	

Abbreviations: CM, chronic migraine; EM, episodic migraine; M + PNB, medications + peripheral nerve block; MIDAS, Migraine Disability Assessment; PNB, peripheral nerve block alone; VAS, Visual Analog Scale.

Notes: ^*^ Wilcoxon signed-rank test; ^#^ Mann-Whitney U test; ^&^ paired-sample *t* test; ^$^ independent-sample *t* test; ^£^ chi-square test.

## DISCUSSION

In the present study, we showed that bilateral GON, LON and SON block performed twice in both EM and CM patients, with an interval of two weeks between applications, was an effective treatment even if the patients only underwent PNB after their drug treatment had been discontinued.

Although PNB has been shown in many studies 4 5 7 10 to be an effective treatment for migraine, its mechanism of action is still not fully understood. It is thought that the inflammation that starts with activation of the trigeminovascular system in migraine attacks causes central sensitization, from which pain and allodynia then develop. 14 Activation of nociceptors in the dura mater and intracranial vessels and central projections of the trigeminocervical complex are also thought to play an important role in pain due to migraine. The GON is a sensory nerve originating predominantly from the C2 segment. Dural afferents and GON afferents establish anatomical connections. The SON is an important branch of the ophthalmic nerve (V1), and it receives the sensory input from the region where migraine pain is most frequently observed. 15 Local anesthetics cause reversible nerve block via voltage-gated sodium channels. It is thought that PNB reduces peripheral stimulation and central sensitization and that it regulates the nociceptive pathway; therefore, it is effective.

Many studies 4 6 16 17 have shown that GON block is an effective treatment in migraine patients. Zhang et al., 5 in a meta-analysis on randomized controlled studies, showed that GON block in migraine patients reduced the severity of pain, number of days with pain and number of analgesics taken, without increasing the risk of side effects.

There is no consensus among previous studies regarding the type of anesthetic agent to be used or whether additional steroids should be used or not. Ashkenazi et al. 17 and Kashipazha et al. 18 found that there was no significant benefit from use of additional steroid. In a study by Malekian et al. 19 among EM patients, using triamcinolone alone and triamcinolone injection plus lidocaine, with a single injection and a one-month follow-up, skin atrophy and alopecia developed in 3 out of 23 patients, who were then excluded. No significant side effects were detected in groups that received only lidocaine and saline injection, and significant superiority of results was found in the groups that received the treatment, in comparison with the placebo group. 19 Use of lidocaine for GON block was therefore suggested. 19 The agents most commonly used as local anesthetics are lidocaine and bupivacaine. Bupivacaine was found to be effective in studies using doses of 1.5 mL and 4.5 mL of 0.5% lidocaine and in studies using doses of 1 mL and 4.5 mL of 2% lidocaine. 17 18 20 21 In the present study, we performed the PNB with 1.5 mL of 2% lidocaine and found that it was an effective treatment without any significant side effects.

There is no consensus on the frequency of the injections among previous studies. It has been reported that the efficacy of treatment is maintained for up to eight weeks in patients who received a single injection. 18 Using a single injection, Dilli et al. 22 found that there was no significant difference in efficacy of treatment, compared with placebo. Ruiz Piñero et al. 11 and Caputi and Firetto 10 reported that patients who did not benefit from a single block benefited from repeated blocks. 11 Karadas et al. 23 compared patients who underwent a single block with those who underwent three blocks once a week and found that the efficacy of treatment, as observed in the 12 ^th^ week, was better in the group that had undergone repeated blocks. In the present study, PNB was performed twice, upon admission and in the second week, and we found that it was an effective treatment, as assessed after two months.

Palamar et al. 24 and Ünal-Artık et al. 25 reported that a single GON block was also effective. Like in most other studies, 5 we performed bilateral PNB.

In many studies, 5 6 17 20 24 GON block alone was found to be effective. Caputi and Firetto 10 reported that there was a significant response in 85% of 23 patients with repeated GON and SON blocks. 10 Ruiz Piñero et al. 11 performed GON and SON blocks according to the origin of pain in 60 patients and reported that 23 of them responded completely in the second week, while 24 patients had a partial response. They performed SON block alone in 18 patients, 13 bilaterally and 5 unilaterally. No separate data were reported regarding the clinical outcomes among the patients who only underwent SON block. 11 In the study by Özer et al., 8 in which bilateral GON and SON blocks and placebo were compared among patients taking prophylactic medications, the proportion of the patients with more than 50% decrease in the number of painful days in the second month was 65.4% in the EM group, 64.7% in the CM group and 28.6% in the placebo group. Thus, the treatments were considered effective. In the present study, we applied SON and LON blocks in addition to GON block, and we found that this formed an effective treatment on its own, with no need for prophylactic medications.

In many studies, 8 10 12 16 GON block was performed in patients who did not respond to drug treatment, and these patients continued to use their medications. We found one study in the literature in which patients who underwent GON block alone and in combination with prophylactic medication treatment were evaluated. In that study, conducted by Inan et al., 9 both groups underwent blocks with weekly 2-mL injection of 0.25% bupivacaine for the first month and monthly thereafter, and the patients were followed up for 3 months. No information was provided regarding the proportions of EM and CM patients. In the third month, GON block was assessed as having been effective in both groups, without any significant differences in terms of the number of days with pain and the severity and duration of pain, between the two groups of patients. 9 The present study was the first to evaluate the effectiveness of PNB in both EM and CM patients who were not taking any drug treatment, and it was the first to assess MIDAS scores and the effectiveness of SON, LON and GON blocks. We found that there was no difference in efficacy of treatment between the two groups. Although the prophylactic medication treatment of the patients who underwent PNB alone was discontinued at least two weeks before the block, the possibility of a residual effect from these drugs was a limiting factor of the present study.

The most important limitations of the present study were its retrospective nature, the absence of a placebo group and the short follow-up. Another limitation was the lack of knowledge of the prophylactic agents that the patients were taking or had discontinued, which may have changed the effectiveness of the local anesthetic agent. The longer duration of diseases in EM M + PNB patients, compared with EM PNB patients, may have affected the results from our study. One the other hand, the most important advantage of the present study was that it was the first to evaluate both EM and CM patients who underwent GON, LON and SON blocks after discontinuation of their drug treatments.

In conclusion, bilateral GON, LON and SON blocks consisting of lidocaine injection seemed to be an effective treatment on its own, with no need for prophylactic medications, in both EM and CM patients during the two-month follow-up. This is an inexpensive and effective treatment option with low risk of side effects, thereby reducing migraine-related disability, which is costly both to patients and to society. There is a need to conduct randomized controlled studies with longer follow-ups and larger samples, in order to assess the duration of the efficacy of treatment, the frequency of its administration and the dose of local anesthetic to be administered.
